# More Evidence on the Impact of India's Conditional Cash Transfer Program, Janani Suraksha Yojana: Quasi-Experimental Evaluation of the Effects on Childhood Immunization and Other Reproductive and Child Health Outcomes

**DOI:** 10.1371/journal.pone.0109311

**Published:** 2014-10-10

**Authors:** Natalie Carvalho, Naveen Thacker, Subodh S. Gupta, Joshua A. Salomon

**Affiliations:** 1 Global Burden of Disease Group and Center for Health Policy, Melbourne School of Population and Global Health, Melbourne, Australia; 2 Center for Health Decision Sciences, Harvard School of Public Health, Boston, Massachusetts, United States of America; 3 Deep Children Hospital and Research Centre, Gandhidham, Gujarat, India; 4 Department of Community Medicine, Mahatma Gandhi Institute of Medical Sciences, Sewagram, Maharashtra, India; 5 Department of Global Health and Population, Harvard School of Public Health, Boston, Massachusetts, United States of America; Stanford University, United States of America

## Abstract

**Background:**

In 2005, India established a conditional cash transfer program called Janani Suraksha Yojana (JSY), to increase institutional delivery and encourage the use of reproductive and child health-related services.

**Objective:**

To assess the effect of maternal receipt of financial assistance from JSY on childhood immunizations, post-partum care, breastfeeding practices, and care-seeking behaviors.

**Methods:**

We use data from the latest district-level household survey (2007–2008) to conduct a propensity score matching analysis with logistic regression. We conduct the analyses at the national level as well as separately across groups of states classified as high-focus and non-high-focus. We carry out several sensitivity analyses including a subgroup analysis stratified by possession of an immunization card.

**Results:**

Receipt of financial assistance from JSY led to an increase in immunization rates ranging from 3.1 (95%CI 2.2–4.0) percentage points for one dose of polio vaccine to 9.1 (95%CI 7.5–10.7) percentage points in the proportion of fully vaccinated children. Our findings also indicate JSY led to increased post-partum check-up rates and healthy early breastfeeding practices around the time of childbirth. No effect of JSY was found on exclusive breastfeeding practices and care-seeking behaviors. Effect sizes were consistently larger in states identified as being a key focus for the program. In an analysis stratified by possession of an immunization card, there was little to no effect of JSY among those with vaccination cards, while the effect size was much larger than the base case results for those missing vaccination cards, across nearly all immunization outcomes.

**Conclusions:**

Early results suggest the JSY program led to a significant increase in childhood immunization rates and some healthy reproductive health behaviors, but the structuring of financial incentives to pregnant women and health workers warrants further review. Causal interpretation of our results relies on the assumption that propensity scores balance unobservable characteristics.

## Introduction

India has some of the worst maternal and child health indicators in the world, with approximately 18% of global maternal deaths and over 20% of all deaths among children under age five years.[1], [2] From 2000 to 2008, India experienced an average annual decline in under-five mortality rate of 3.9%, with highly uneven progress across states, and falling short of the 4.4% reduction per year required to meet the 2015 Millennium Development Goal (MDG) 4 target.[3], [4], [5] Since 2008, India has experienced a higher rate of decline in under-five mortality,[6] some of which may be attributed to the launch of the national rural health mission in 2005. India now appears much closer to achieving MDG4, which looked distant a few years ago.

Childhood immunizations are critical to safeguarding child health. According to the World Health Organization (WHO), approximately 40% of all under-vaccinated children, defined as children who did not receive 3 doses of diphtheria, tetanus and pertussis (DPT) in their first year of life, live in India.[7] Although immunization rates have increased over time, only slightly more than half of children nationwide are fully vaccinated, with wide variations across geographic and socioeconomic strata.[8] Inadequate rates of childhood immunization persist despite vaccinations being provided free of charge in public health facilities through India's Universal Immunization Program (UIP), which covers 27 million infants and 30 million pregnant women annually.[9], [10]

In 2005, India's Ministry of Health and Family Welfare (MOHFW) launched the National Rural Health Mission (NRHM), which aimed to bring in health sector reforms to strengthen public health management and ensure effective health care delivery.[11], [12] A key feature of NRHM is a safe motherhood scheme called Janani Suraksha Yojana (JSY). JSY is a conditional cash transfer program that provides financial incentives to pregnant women and female community health workers to encourage the use of health services during the antenatal, intrapartum and post-partum period.[11] With a goal of reducing maternal and childhood mortality, JSY aims to increase safe deliveries among women of low socioeconomic status by promoting institutional deliveries, especially in rural areas. The program operates at the community level through an accredited social health activist (ASHA) who is selected by NRHM to act as the intermediary between the women and the state. ASHAs are responsible for identifying all pregnant women in their community and facilitating their use of reproductive health services offered by the state, including antenatal care visits, facility-based delivery, postnatal checkups, immunization of the newborn, and providing advice and counseling on breastfeeding practices.[13]

Janani Suraksha Yojana is a national program funded exclusively by the federal government and managed at the local level by states.[14], [15] Eligibility criteria and financial incentives vary across states, and have been modified over time. In ten Low Performing States (LPS) (Assam, Bihar, Chhattisgarh, Jammu and Kashmir, Jharkhand, Madhya Pradesh, Orissa, Uttarakhand, and Uttar Pradesh) with low rates of institutional deliveries, all women are eligible for the program. These states also tend to have higher fertility rates and worse maternal and child health indicators compared to the rest of the country.[14] Among the other High Performing States (HPS), eligibility is restricted to marginalized women (those with a government-issued below the poverty line (BPL) card or belonging to a scheduled caste or tribe), and only for their first two births.[15] Eligible women receive cash assistance ranging from 600 Indian rupees (Rs.) (∼US $10 as of 2014) in urban areas of HPS to 1,400 Rs. (∼US $23) in rural areas of LPS upon delivering in an accredited facility.[15] BPL women continue to receive 500 Rs. (∼US $8) for deliveries outside of health facilities for their first two births.[16]

Previous studies have shown that JSY led to increased institutional deliveries.[15], [17], [18] An impact evaluation carried out across all states and union territories (UTs) using three different analytical approaches found a small but significant effect of JSY on increasing antenatal care and reducing perinatal and neonatal mortality among two of three analytic approaches.[17] A more recent impact evaluation, carried out using the same data, found little to no impact of JSY on antenatal care and did not find a significant impact on neonatal and perinatal mortality.[18] Among other methodological differences between the two studies, the latter's differences-in-differences analysis accounts for heterogeneity in timing of the introduction of JSY across districts, in order to control for potential unobserved district-level confounders.[18] Mazumdar and colleagues' preferred estimates were able to statistically rule out a reduction in neonatal mortality of greater than 8.7 deaths per 1,000 live births.[18] In comparison, estimates of the effect of JSY from a matching analysis by Lim et al. indicated a reduction of 2.3 neonatal deaths per 1,000 live births.[17] Lim's district-level differences-in-differences estimates of the effect of JSY on health outcomes showed no statistically significant effect on neonatal or early neonatal mortality.[17] However, Lim and colleagues note that this analysis may not have been powered to detect the reduction in perinatal and neonatal mortality found through their other analytical approaches.[17]

While childhood vaccinations were not the main target of JSY, the program could have had a direct or indirect effect on these outcomes. Early guidelines indicated minimum payments to ASHAs per in-facility delivery of 200 Rs. (∼US $3) in urban areas and 600 Rs. in rural areas of LPS, north-east states, and tribal areas, with disbursement provided in two payments, the first upon reaching the institution along with the expectant mother, the second after making a postnatal visit and the child has been immunized with the bacillus Calmette-Guerin vaccine (BCG).[16] More recent government documents indicate financial incentives provided to ASHAs upon (1) motivating women to seek institutional delivery and antenatal care, (2) payment for transport of the pregnant woman to a facility, and (3) escort of the pregnant woman to the institution.[19] Aside from incentives, increased interaction with the health system through institutional deliveries could indirectly lead to an increase in childhood immunizations. Although there is some evidence that immunization rates have increased following the start of JSY, there has been no formal evaluation of the impact of JSY on childhood immunization rates.[15], [20] Prior studies evaluating the effect of conditional cash transfers on immunization coverage have generally found minimal improvements in vaccination coverage or non-significant results.[21] The majority of evidence comes from Latin America, and in most study areas, vaccination coverage was high prior to the program's start. A recent study of cluster randomized controlled campaigns in a setting with low immunization coverage in India found that providing small non-financial incentives with improvements in the reliability of services led to a large increase in immunization rates, at a cost of approximately $17.35 per child.[22]

This study evaluates the impact of JSY on childhood immunization rates in India. Using a quasi-experimental analytic design, we compared childhood immunization outcomes among women who had received financial assistance from JSY compared to those who had not, controlling for possible confounders. We also evaluated the impact of the program on a range of secondary outcomes, including receipt of postnatal care, breastfeeding practices, and care-seeking behavior in the post-natal period, using the same approach.

## Methods

### Study design and participants

We conduct a multivariable logistic regression, with matching to control for confounding, to compute the average effect of JSY on reproductive and childhood outcomes. Many of the analyses were modeled after the matching analytical approach used by Lim et al.[17]

Data used were from the most recent round of the District Level Household Survey (DLHS-3), one of the largest demographic and health surveys conducted in India. The DLHS-3 is a nation-wide survey designed primarily to provide estimates of reproductive and child health indicators.[8] The survey was carried out across 34 states and union territories in India (excluding Nagaland) from December 2007 to December 2008. Using a multi-stage stratified sampling design, interviewers collected data from 720,320 households across urban and rural areas of 601 districts in the country.[8] For households in rural areas, a village-level questionnaire covering 22,825 villages was used to gather information on village characteristics. We used responses from currently married women aged 15–44 years who reported having had a live birth within the period covered by the survey (from January 1, 2004 onwards).

In analyses on postnatal care, breastfeeding practices, and care-seeking behaviors, data were restricted to children born within the last 12 months before the survey, to obtain the most recent sample, and to reduce the effect of varying fertility rates and differential introduction and scale-up of JSY, on study results.[17], [18] For analyses on immunizations, data were restricted to children 12-23 months of age who were alive at the time of the interview to prevent premature censuring of vaccine outcomes among children under 12 months of age.[23]

This study was approved by the Population and Global Health Human Ethics Advisory Group at the Melbourne School of Population and Global Health, University of Melbourne.

### Study measures

Women were asked whether they had received financial assistance from JSY for their most recent delivery; those who responded “yes” to this question were coded as JSY  =  1, and those who responded “no” were coded as JSY  =  0. Because women were only asked about their most recent pregnancy, only data on women's most recent live birth could be used to investigate the effect of JSY on post-delivery indicators and immunization rates.

Childhood immunization outcomes considered include receiving the following vaccines: polio at birth (or “polio zero”), one dose of BCG, at least one dose of DPT, three doses of DPT, at least one dose of polio, three doses of polio, measles, and any hepatitis B.[24] We also considered the proportion of fully vaccinated children and children who did not receive any vaccination. In line with WHO guidelines we defined a fully vaccinated child as one who had received one dose of BCG vaccine, 3 doses of DPT and polio vaccines (not including polio at birth), and one dose of measles vaccine by the age of 12 months.[8], [25]

Vaccination status was determined from immunization cards, supplemented by mothers' reports where immunization cards were incomplete or missing. While there are important limitations to using this type of data, household surveys are regularly used for estimating childhood immunization rates.[23] For polio at birth, we include children who had an immunization date for polio zero in their immunization cards, and those whose mothers reported them having their first polio vaccine within 2 weeks of birth. Information on hepatitis B was not included in the immunization card data; it was the only immunization outcome that relied solely on maternal reporting. Furthermore, although three doses of hepatitis B are recommended (similar to DPT and polio),[24] only one survey question was asked about any hepatitis B vaccination.

Children with missing data (<0.5% of observations) or with a response of “don't know” reported for one or more vaccines were treated as missing observations, and these children were not included in the denominator. We also considered a more conservative definition for all vaccines, counting children with missing vaccination data or “don't know” responses as not having been vaccinated; this more conservative definition matched immunization means reported in the DLHS-3.[8]

Other reproductive and child health indicators included prompt post-natal check-ups for the mother (within 48 hours of delivery) and baby (within 24 hours of delivery), three breastfeeding behavior outcomes (early initiation of breastfeeding within the first hour of birth, child breastfed colostrum, exclusively breastfed for 6 months or continuing to be breastfed), and care-seeking behaviors for symptoms of childhood diarrhea and pneumonia (sought advice or treatment).

Other measures available from the household survey and included as covariates in the analysis were measures of household assets, maternal age and education, information on birth history, gender of the child, caste or tribe, religion, below-the-poverty-line card ownership, urban or rural residence, and distance to the nearest health facility.

### Statistical Analysis

We used factor analysis to construct a household wealth index based on the following categorical household characteristics and assets: access to an improved drinking water source; access to improved sanitation; type of house (3 categories, with pucca of highest quality, kaccha of lowest quality); type of cooking fuel; access to an electricity connection; presence of other household assets including fan, television, telephone, scooter and car. Household wealth quintiles and deciles were generated from this wealth index.

To investigate the effect of maternal receipt of financial assistance from JSY on childhood immunization rates and other reproductive and child health indicators, we conducted a propensity-score matching (PSM) analysis with logistic regression to control for potentially confounding differences between the JSY and non-JSY groups. PSM is a widely used method in impact evaluation literature when experimental data are not available. This method can correct for biases in treatment effect due to observed covariates, that result from confounding due to non-random assignment of the treatment.[26] Matching allows for the “treated” group to be made as similar to the “untreated” group as possible based on observed pre-treatment matching covariates, to reduce the link between the treatment variable (receipt of JSY) and background characteristics of the participant.[27] In order to draw causal inferences, this method relies on the assumption that balancing observables also balances unobservables.

We used a logit model to estimate propensity scores and 1:1 nearest neighbor matching algorithm without replacement to generate matched groups. Matching covariates included maternal age, number of live births, birth interval, whether the birth was part of a multiple birth, maternal education category, household wealth decile, BPL-card ownership, caste or tribe, religion, location of residence with respect to distance to the nearest health facility, and state of residence. We defined categorical variables to be consistent with the Lim et al. analysis,[17] to facilitate comparison to prior findings. We performed several PSM diagnostics including visual inspection of propensity scores in the treated and control group pre- and post- matching and comparison of background characteristics between groups pre- and post-matching. (**Figure S1** and **Table S1**)

The main analysis to identify ‘treatment effects’ for JSY used logistic regression with state-level fixed effects and robust, clustered standard errors at the district level. We included the same regression covariates as in the PSM step. The estimated treatment effect for a given outcome was obtained using fitted probabilities, by computing the difference between the probability of the outcome of interest for the treated group (JSY  =  1) and the control group (JSY  =  0), which results in the interpretation of the findings as average effects across all JSY recipients. We repeated analyses separately for LPS and HPS. Following prior guidance suggesting that survey weights are not needed for matched analyses if the model is correctly specified,[28] we did not include survey weights in the main analysis, but we conducted a sensitivity analysis that did include survey weights. The propensity score matching was done in R (version 2.12.1) and all other analyses were conducted in Stata (version 12).

### Sensitivity analyses

We tested various alternative model specifications such as including child's gender and an indicator for LPS (alone and interacted with the treatment effect) as covariates, running the analysis with district-level fixed effects, and accounting for calendar time heterogeniety. We carried out an analysis including ever-married women 45–49 years of age. For immunization outcomes, we replicated the coarsened-exact matching analysis carried out by Lim and colleagues using the same coarsened matching covariates as the authors.[17] For these outcomes, we also re-ran the analyses restricted to children born within the last 12 months before the survey. In addition, we reran the analyses separately for individuals with immunization cards and those missing immunization cards, to investigate any potential differences in the effect of JSY between the two groups. Roughly 43% of individuals had immunization cards for their children that they were able to produce to the interviewers. Of the remaining 57% of the sample, just over half (54%) reported having an immunization card but were unable to show it to the interviewers, and the remaining group did not have a card. We grouped together all families that were unable to show an immunization card to the survey interviewers (and thus relied on parental recall) and considered them as those with missing immunization cards. Finally, we re-ran the propensity score matching and logistic regressions separately for women who delivered in a health facility, and women who delivered elsewhere. While susceptible to endogeneity bias, this stratified analysis allows for the control of unobserved heterogeneity between women who delivered in facilities and those who didn't, and corrects for biases related to the potential reverse causality between institutional delivery and receipt of the cash transfer.[18]

## Results

Mean immunization rates among our sample population (children aged 12 to 23 months that were the most recent births of women 15 to 44 years of age) are shown in **Table 1**. Nearly 95% of children had been vaccinated at least once against polio, while only 71% of children had received a measles vaccine. Large drops in coverage rates between the first and third recommended doses were seen for both polio (23 percentage point drop) and DPT (18 percentage point drop) vaccines. At the national level, 54% of children aged 12 to 23 months were fully vaccinated; less than 5% of children had received no vaccine.

**Table 1:** National level immunization coverage estimates, most recent births 12–23 months prior to survey to women 15–44 years.

	Mean	95% CI, upper	95% CI, lower
BCG	87.5%	87.2%	87.8%
Polio at birth	58.1%	57.6%	58.5%
Polio 1	93.8%	93.5%	94.0%
Polio 3	70.9%	70.4%	71.3%
DPT 1	83.9%	83.6%	84.2%
DPT 3	66.0%	65.6%	66.4%
Measles	70.9%	70.5%	71.3%
Hepatitis B	29.6%	29.2%	30.1%
Fully vaccinated child*	54.1%	53.7%	54.6%
No vaccine	4.6%	4.5%	4.8%

*A fully vaccinated child was defined as a child who had received one dose of BCG vaccine, 3 doses of DPT and polio vaccines (not including polio at birth), and one dose of measles vaccine. [IIPS 2010]

**Figure 1:**
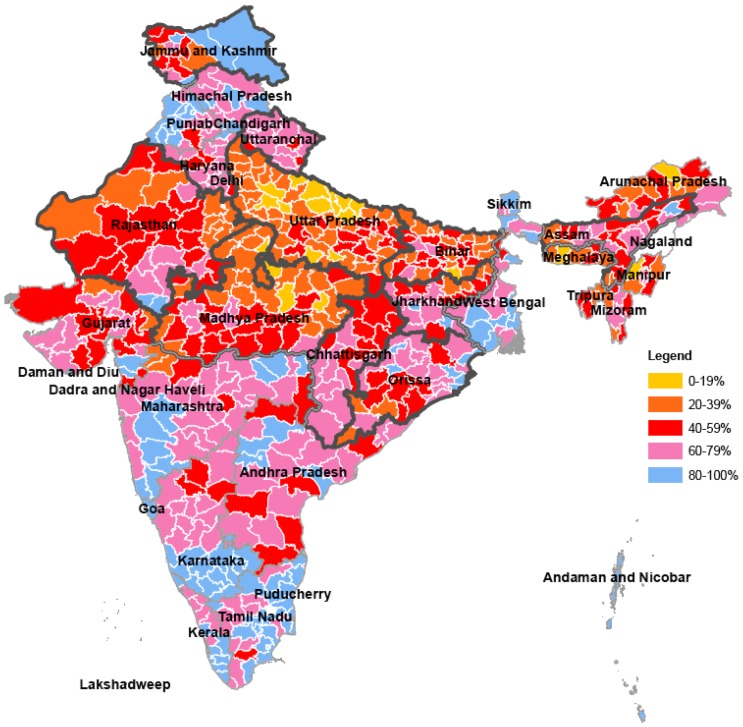
Percent of children 12–23 months at the time of the survey who were fully vaccinated by district, for high and low performing states, 2007–08. * *Among most recent births for women ages 15–44 years of age. A fully vaccinated child was defined as a child who had received one dose of BCG vaccine, 3 doses of DPT and polio vaccines (not including polio at birth), and one dose of measles vaccine. [IIPS 2010] Dark (bolded) outlines represent the ten low-performing states (LPS): Uttar Pradesh, Uttarakhand, Bihar, Jharkhand, Madhya Pradesh, Chhattisgarh, Assam, Rajasthan, Orissa, and Jammu and Kashmir. Districts with no data are in white.

National-level means mask substantial variation in immunization rates across geographic and socioeconomic strata of the country. **Figure 1** shows district-level variations in the proportion of children aged 12 to 23 months who are fully vaccinated. States with bolded outlines are LPS. As can be seen from this figure, these LPS, along with the Northeast states, both of which were priorities of JSY, have consistently lower immunization rates compared to HPS. (**Figure 1**)

Selected results from the multivariate logistic regression on matched samples are shown in **Table 2**. (Full regression results in **Table S2**) Computed predicted probabilities show that receipt of financial assistance from JSY led to a significant increase in immunization rates of several percentage points among children aged 12–23 months, across all vaccines considered. (**Table 3**) With the exception of hepatitis B, which was borderline significant at the 95% confidence level in the base case analysis, the smallest effect of JSY (3.1 percentage points) was on the first dose of polio vaccine, which also had the highest national coverage rate (94%). (**Table 1**) The largest effect sizes (7.8 percentage points) were seen on the coverage of polio zero and DPT3, which have much lower national level coverage rates. (**Table 1**) For most vaccines, JSY payments resulted in a 3 to 8 percentage point increase in coverage. Maternal receipt of cash payments from JSY led to an increase in 9.1 percentage points in the proportion of fully vaccinated children, and a reduction of 3.2 percentage points in the proportion of children who had not received a single vaccine.

**Table 2:** Abbreviated regression results for selected immunization outcomes, run on sample of children 12–23 months of age.

		BCG n = 12,520			Fully vaccinated* n = 12,592			Not Vaccinated n = 12,177		
		OR	SE	p-value	OR	SE	p-value	OR	SE	p-value
	JSY	2.57	0.22	<0.01	1.58	0.06	<0.01	0.36	0.04	<0.01
Maternal age (years)	15–19	0.65	0.13	0.04	0.72	0.08	<0.01	2.11	0.64	0.01
	20–24	0.74	0.12	0.06	0.78	0.06	<0.01	2.62	0.59	<0.01
	25–29	0.95	0.14	0.70	0.89	0.07	0.11	1.98	0.43	<0.01
	30–34	1.00	–	–	1.00	–	–	1.00	–	–
	35–39	0.87	0.19	0.53	0.85	0.11	0.21	1.57	0.48	0.14
	40–44	1.47	0.51	0.27	0.86	0.20	0.51	0.35	0.25	0.14
Number of live births	1 birth	1.00	–	–	1.00	–	–	1.00	–	–
	2 births	0.98	0.11	0.89	0.86	0.05	<0.01	1.09	0.15	0.55
	3–4 births	0.74	0.09	0.01	0.72	0.05	<0.01	1.55	0.25	0.01
	5 or more	0.54	0.12	<0.01	0.47	0.06	<0.01	2.33	0.65	<0.01
Maternal education	No education	1.00	–	–	1.00	–	–	1.00	–	–
	1–5 years	1.41	0.16	<0.01	1.35	0.08	<0.01	0.71	0.10	0.02
	6–11 years	2.34	0.27	<0.01	1.73	0.09	<0.01	0.45	0.07	<0.01
	12 or more	2.72	0.76	<0.01	1.77	0.17	<0.01	0.37	0.15	0.02
Household wealth	Poorest decile	1.00	–	–	1.00	–	–	1.00	–	–
	Decile 2	0.93	0.13	0.60	1.12	0.10	0.21	0.82	0.17	0.34
	Decile 3	1.24	0.19	0.15	1.14	0.10	0.13	0.89	0.16	0.54
	Decile 4	1.08	0.16	0.62	1.23	0.11	0.02	0.89	0.18	0.56
	Decile 5	1.36	0.22	0.06	1.27	0.11	0.01	0.69	0.14	0.08
	Decile 6	1.47	0.26	0.03	1.27	0.12	0.01	0.63	0.14	0.04
	Decile 7	1.49	0.28	0.03	1.48	0.14	<0.01	0.35	0.10	<0.01
	Decile 8	1.97	0.42	<0.01	1.74	0.18	<0.01	0.33	0.10	<0.01
	Decile 9	2.98	0.86	<0.01	1.91	0.23	<0.01	0.29	0.12	<0.01
	Richest decile	14.25	9.04	<0.01	2.27	0.35	<0.01	0.14	0.09	<0.01

* A fully vaccinated child was defined as a child who had received one dose of BCG vaccine, 3 doses of DPT and polio vaccines (not including polio at birth), and one dose of measles vaccine. [IIPS 2010]

**Table 3:** National level results from logistic regression of JSY effects on immunization outcomes among most recent births 12–23 months prior to survey to women 15–44 years.

	Estimated JSY treatment effect			N
	Point est.	95% CI, lower	95% CI, upper	
BCG	4.9%	4.0%	5.8%	12,520
Polio at birth	7.8%	6.1%	9.4%	12,303
Polio 1	3.1%	2.2%	4.0%	12,526
Polio 3	6.3%	5.0%	7.6%	12,026
DPT1	5.6%	4.6%	6.6%	12,436
DPT3	7.8%	6.3%	9.3%	12,188
Measles	5.9%	4.4%	7.3%	12,438
Hepatitis B	1.8%	0.3%	3.3%	11,907
Fully Vaccinated*	9.1%	7.5%	10.7%	12,592
No vaccine	−3.2%	−4.0%	−2.4%	12,177

*A fully vaccinated child was defined as a child who had received one dose of BCG vaccine, 3 doses of DPT and polio vaccines (not including polio at birth), and one dose of measles vaccine. [IIPS 2010]

A conservative definition of vaccine status, which considered children with missing or “Don't know” responses as not having been vaccinated, produced the same results across all vaccines, with the exception of polio at birth. For this outcome, which had high proportion (8%) of “Don't know” responses, particularly from caregivers asked whether their child had received their first polio vaccine within 2 weeks of birth, the estimated JSY treatment effect was lower than that found using the base case vaccine status definitions.

National-level means for all other reproductive and child health indicators, including postnatal check-up rates, breastfeeding behavior, and IMCI-related indicators are shown **Table 4**. Nearly half of all mothers and their newborns received a postnatal check-up following delivery. While the majority of mothers (82%) breastfed colostrum to their baby, less than half (41%) started breastfeeding within one hour of birth. Only 37% of infants born within the last 12 months prior to the survey were exclusively breastfed for 6 months (or were still currently being breastfed). The majority of caregivers sought advice or treatment if their children had diarrhea, fever, or symptoms of pneumonia.

**Table 4:** National level child health outcomes relating to the most recent births to women 15–44 years born within the last 12 months prior to the survey.

	Mean	95% CI, upper	95% CI, lower
*Post-natal care*			
Woman, within 48 hr after delivery	49.9%	49.4%	50.3%
Newborn, within 24 hr after birth	50.6%	50.1%	51.0%
*Breastfeeding behavior*			
Early initiation of breastfeeding*	41.1%	40.7%	41.5%
Breastfed colostrum to child	81.2%	80.9%	81.5%
Excl. breastfed for 6 months or continuing	37.0%	36.6%	37.4%
*IMCI* ** *indicators*			
Sought advice or treatment for diarrhea	68.5%	67.5%	69.5%
Sought advice or treatment for symptoms of pneumonia*** or fever	73.1%	72.3%	73.8%

*Defined as started breastfeeding within 1 hour of birth.

**Integrated management of childhood illnesses (IMCI).

***Pneumonia defined as cough plus fast breathing.

Receipt of financial assistance from JSY had a large and significant positive effect of 26–27 percentage points on postnatal check-ups among mothers and newborns. (**Table 5**) JSY also had a positive effect on breastfeeding behaviors immediately following childbirth. Of 100 women who received cash assistance from JSY, an additional 7 women began breastfeeding within an hour after delivery, and an additional 4 women breastfed their baby colostrum. No significant effect was found from maternal receipt of financial assistance from JSY on exclusive breastfeeding or care-seeking behaviors for sick children.

**Table 5:** National level results from logistic regression of JSY effects on child health outcomes among most recent births to women 15–44 years born within the last 12 months prior to the survey.

	Estimated JSY treatment effect			N
	Point est.	95% CI, lower	95% CI, upper	
*Post-natal care*				
Woman, within 48 hr after delivery	24.8%	22.9%	26.7%	24,258
Newborn, within 24 hr after birth	25.7%	23.9%	27.4%	23,924
*Breastfeeding behavior*				
Early initiation of breastfeeding*	6.8%	5.3%	8.3%	23,923
Breastfed colostrum to child	4.1%	3.0%	5.2%	23,917
Excl. breastfed for 6 months or continuing	−1.0%	−2.5%	0.4%	23,316
*IMCI* ** *indicators*				
Sought advice or treatment for diarrhea	3.7%	0.6%	6.9%	3,754
Sought advice or treatment for symptoms of pneumonia*** or fever	2.0%	−0.2%	4.3%	5,799

*Defined as started breastfeeding within 1 hour of birth.

**Integrated management of childhood illnesses (IMCI).

**Figure 2:**
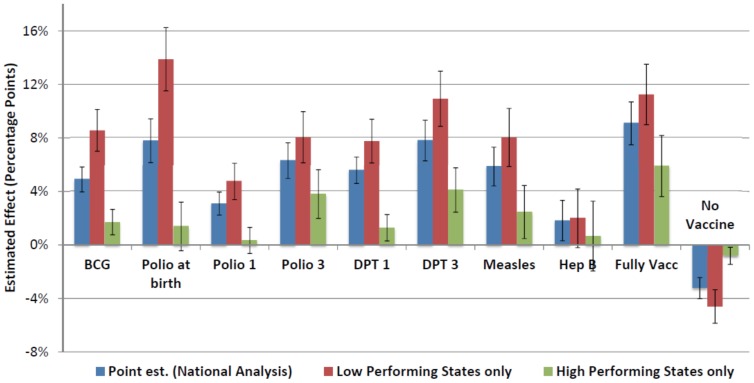
Estimated JSY treatment effect on childhood immunization outcomes among children 12 to 23 months of age, stratified by LPS and HPS compared to national level results. Error bars represent 95% confidence intervals from regression estimates. * A fully vaccinated child was defined as a child who had received one dose of BCG vaccine, 3 doses of DPT and polio vaccines (not including polio at birth), and one dose of measles vaccine. [IIPS 2010]

Results were consistent across an array of different model specifications. Hepatitis B was the only exception, for which the treatment effect ceased to remain significant across several robustness checks. Effect estimates were insensitive to the use of survey weights and calendar time of interview fixed effects. Child's gender was found to have a small but significant association with postnatal check-ups, seeking advice or treatment for diarrhea or pneumonia, and some vaccination outcomes, with male children slightly more likely to be involved in these healthy behaviors. Being a LPS was negatively associated with all reproductive and child health indicators considered, controlling for individual-level covariates, and this association was significant across all outcomes except care-seeking behaviors. When including interaction effects between LPS and JSY, the treatment effect of JSY ceased to remain significant for some immunization outcomes (polio zero, first dose of polio, no vaccine) and the early breastfeeding outcomes. For these outcomes, the differential effect of the program in LPS remained significant. Similarly to findings by Lim et al., the treatment effect of JSY was larger in LPS across all immunization outcomes (**Figure 2**) and all check-up and breastfeeding behavior outcomes considered.[17] Including district-level fixed effects had minimal impact on results for most vaccine outcomes. The estimated treatment effect had overlapping 95% confidence intervals for all but the “no vaccine” outcome, for which the effect size was larger (−6.9% vs −3.2%) compared to the base case. Results from the coarsened exact matching method were similar to the base case findings. (**Figure S2**)

**Figure 3:**
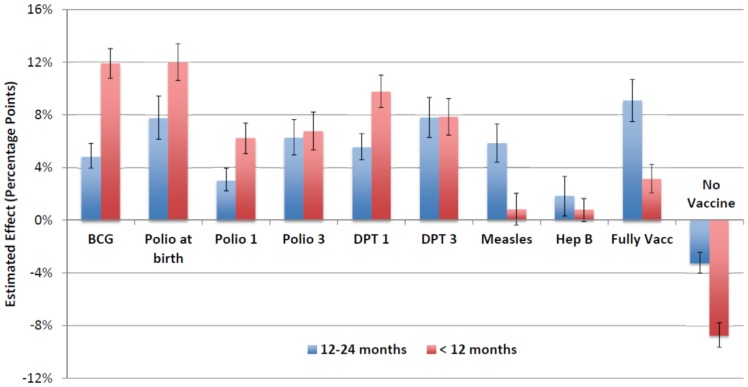
Estimated JSY treatment effect on childhood immunization outcomes: among children 12 to 23 months of age and children under 12 months of age. Error bars represent 95% confidence intervals from regression estimates. * A fully vaccinated child was defined as a child who had received one dose of BCG vaccine, 3 doses of DPT and polio vaccines (not including polio at birth), and one dose of measles vaccine. [IIPS 2010]

**Figure 4:**
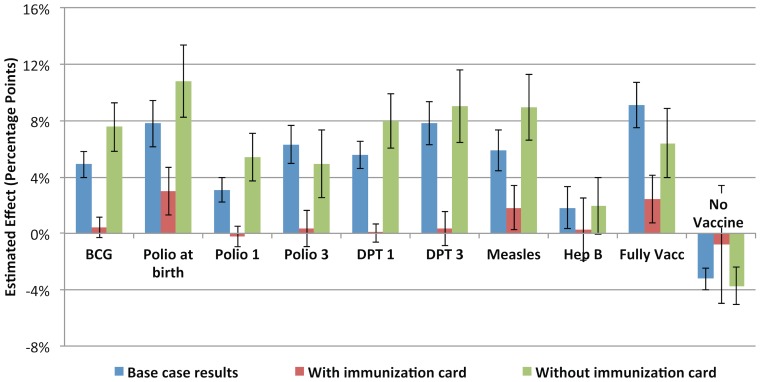
Estimated JSY treatment effect on childhood immunization outcomes among children 12 to 23 months of age: stratified by possession of an immunization card. Error bars represent 95% confidence intervals from regression estimates. * A fully vaccinated child was defined as a child who had received one dose of BCG vaccine, 3 doses of DPT and polio vaccines (not including polio at birth), and one dose of measles vaccine. [IIPS 2010]

**Figure 5:**
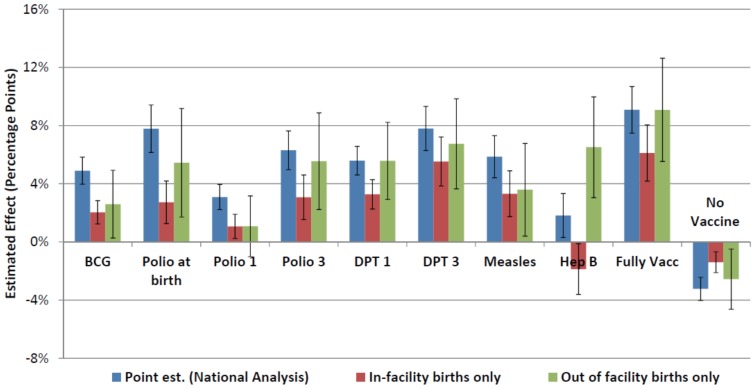
Estimated JSY treatment effect on childhood immunization outcomes among children 12 to 23 months of age: nationally and stratified by delivery location. Error bars represent 95% confidence intervals from regression estimates. * A fully vaccinated child was defined as a child who had received one dose of BCG vaccine, 3 doses of DPT and polio vaccines (not including polio at birth), and one dose of measles vaccine. [IIPS 2010]

In the sensitivity analysis restricted to most recent births within the last 12 months prior to the survey, larger effects of JSY were found among vaccine outcomes that occur close to the time of birth (including no vaccine), while smaller effects were found for measles and the proportion of fully vaccinated children. (**Figure 3**) Because most children in this sample population are under 12 months of age, their vaccine status would be subject to a censoring effect. This effect would be greatest for vaccine outcomes that occur closer to 1 year of age (measles, third dose of polio and DPT, fully vaccinated). Results from the analysis stratified by possession of an immunization card showed important differences in the effect of JSY across both groups. Receipt of financial incentives from JSY had a small (≤ 3%) or no effect among those with vaccination cards while the effect size was much larger than the base case results for the group missing vaccination cards, for nearly all immunization outcomes. (**Figure 4**) For all outcomes, mean immunization levels were consistently higher in the group with vaccination cards. (**Table S3**) Finally, a stratified analysis by delivery location generally resulted in lower treatment effect sizes, particularly among analyses restricted to women delivering in a health facility. (**Figure 5**) Most results remained significant at the 95% confidence level despite much wider confidence intervals, especially among out-of-facility deliveries that involved smaller sample sizes.

## Discussion

Our results indicate that India's conditional cash transfer program led to improvements in reproductive and child health indicators in India, in particular childhood immunization outcomes. Receipt of cash assistance for delivery resulted in increased immunization rates, by several percentage points, across the full range of vaccines considered. The smallest effect was seen in a single dose of polio vaccine, which had a coverage rate of nearly 95%. Vaccines with the lowest coverage rates (polio at birth, three doses of DPT and polio, and measles) had higher treatment effects, ranging from six to eight percentage points increase. The treatment effect of JSY on the proportion of children 12 to 23 months of age who were fully vaccinated was an increase of nine percentage points. In other words, for every 100 children whose mother received financial assistance from JSY for delivery, nine additional children were fully vaccinated. The effect size for hepatitis B immunization was small, and ceased to remain significant across robustness checks. It is worth noting that hepatitis B vaccine was introduced into select states and districts as a pilot in 2002–03, and only expanded to the rest of the country in 2010–11.[9] Although women received cash assistance from JSY at the time of delivery, as opposed to when their child was vaccinated, the effects on immunizations were still found to be significant, and for some vaccines, quite large. Similarly, Lim et al. had noted a significant effect of JSY on increasing antenatal care, for which payment was not directly linked.[17]

There are several mechanisms through which cash transfers for safe deliveries could impact on post-delivery reproductive and child health indicators. One hypothesis is that increased interaction with the health system as a result of JSY could have add-on effects on health-related behaviors, particularly in the early post-partum period. Although some have hypothesized that childhood vaccinations occur too far after delivery for JSY to have an impact,[18] several vaccinations, such as polio at birth and BCG, take place at the time of childbirth or soon afterward. In addition, the role of ASHAs involves promoting healthy reproductive behaviors in the postpartum period including immunizations. In a qualitative assessment carried out in eight LPS states, it was found that although ASHAs were not provided additional incentives for postnatal visits, they made more home visits than any other grassroots functionary.[19] In assessments carried out in 5 LPS, the majority of ASHAs surveyed responded that they had provided help or advice regarding breastfeeding practices and had recommended childhood vaccinations to pregnant women and recently delivered mothers.[15], [29] A small study using records from a tertiary level health center in the state of Orissa found that ‘at birth’ immunization (within 7 days of birth) increased significantly following the implementation of JSY at the health center.[30] In this study, ASHAs were cited by parents as the primary motivator for immunization.

Still, it remains unclear just how much influence ASHAs have on women's behavior, especially decisions outside of choosing an institutional delivery.[31] A few schemes started in the last two years are expected to create more opportunities for ASHAs to reach families during the postnatal period. In a scheme launched in 2011 to strengthen home-based newborn care (HBNC), ASHAs will be given incentives for providing home visits, with incentives tied to BCG at birth and DPT and polio vaccines at 6 weeks.[32] Another scheme exists for home delivery of contraceptives through ASHAs, which will also help to increase contact during the postnatal period.[33] Although incentives are not tied with breastfeeding under this scheme, early breastfeeding is an indicator for monitoring the HBNC scheme. Also, the training package for ASHAs incorporates knowledge on immunization and child nutrition. These approaches create more opportunities for postnatal home visits by ASHAs, and may help to increase immunization coverage and improve breastfeeding and complementary feeding practices in India.

The large impact of JSY on postnatal check-ups was expected given JSY has previously been found to have led to a considerable increase in institutional deliveries, but offers a useful validation of the model and analysis. Perhaps more surprising was the minimal effect on breastfeeding behaviors. Although an increase in several percentage points was seen for early initiation of breastfeeding and among children breastfed colostrum, no effect of cash assistance for delivery was found on exclusive breastfeeding rates, even though the proportion of children who were exclusively breastfed for 6 months or currently being breastfed was well under 50%. Qualitative evidence from surveys conducted in five LPS showed that while ASHAs responded similarly to questions asking about type of support or advice provided to pregnant women or recently delivered mothers regarding immunizations and breastfeeding behaviors, responses from recently-delivered women indicated less advice received from ASHAs regarding breastfeeding behaviors compared to immunizations.[15], [29]

Results remained consistent across a range of model robustness checks and sensitivity analyses. However, there are several important limitations to consider. First, this analysis uses propensity score matching on non-experimental data to make causal claims. Doing so relies on the assumption that balancing observations based on observable characteristics also balances unobservables. This is a strong assumption that we were unable to test. Although we have matched observations on a number of individual and household level covariates that are likely to affect receipt of financial assistance from JSY and the outcomes of interest, our results are not robust against bias arising from unobservable characteristics that are correlated with uptake of JSY and study outcomes. Another main limitation of this analysis concerns the differential timing and scale-up of JSY across the country. While the program was officially established by the federal government in April 2005, it took months (and in some cases over a year) for JSY to be implemented and operationalized across all states and UTs. During implementation of JSY, priority was given to low performing states and the scheme was launched early, while in several high performing states, ASHA recruitment was slow and did not cover all areas during the first few years of the scheme. Furthermore, differences in how the scheme was institutionalized, including the way JSY was advertised, the effectiveness of ASHAs, and the paperwork required for eligibility, could have led to important differences in the effectiveness of JSY across states and districts.

The survey data used for this analysis cover the period soon after JSY was established, and thus the effects may differ compared to when implementation is complete and awareness of the scheme is high across all states. Restricting the analysis to most recent births in the last 12 months reduces issues related to the differential introduction of JSY across districts and states: the earliest births included in analyses restricted to this sample population occurred in December 2006, over 1.5 years after JSY was established. To avoid censored observations as a result of partially immunized children, analyses on vaccination outcomes were restricted to children 12 to 24 months of age. This sample thus included children born seven months to over 2.5 years after JSY was established. We explored including an indicator for LPS to potentially reduce any additional bias due to heterogeneous timing in the implementation of JSY across the country. While this was only a partial remedy, any bias in the treatment effect of JSY will be towards the null, and the estimates obtained will likely underestimate the true effect of JSY. As an additional check, our breastfeeding results are similar to those found by Mazumdar and colleagues of a statistically significant effect of JSY of 7.4 percentage points on breastfeeding in the first hour, but no effect on breastfeeding behavior within 24 hours.[18] Their analysis controlled for time invariant district-level unobservables and accounted for heterogeneity in the timing of the introduction of JSY across the country.[18]

The validity of our results is limited by the quality of the household survey data, in particular the reliability of immunization card data and maternal recall and self-reporting on their child's vaccination status. The subsample analysis shows that these two groups differ significantly with respect to the effect of JSY on childhood immunization outcomes. Maternal receipt of financial incentives from JSY had little to no effect among the group with immunization cards. There are several possible explanations for this. First, it is likely that these two groups are systematically different from each other in ways that are not controlled for in the analysis. Second, immunization rates among the group relying on maternal recall may be over- or underestimated. It is also possible that those relying on maternal recall may be more likely to report receiving specific vaccinations linked to the JSY program even if their child had not received the vaccine. In this case, the effect of the program would be overestimated. On the other hand, childhood immunization rates are much higher among those with immunization cards, with little room for increased coverage for some vaccine outcomes.

We also did not consider the timing of vaccines and whether vaccines were received at the appropriate time to ensure full protection against disease.[34] Interestingly, restricting the sample to children born in the last 12 months prior to the survey generally resulted in larger treatment effects among immunization outcomes that occur early in life, possibly indicating improved timing of vaccines with JSY.

In an analysis stratified by in-facility delivery, we attempted to control for unobserved heterogeneity between women who delivered in facilities and those who did not. While treatment effects are lower in both stratified analyses, we still find significant effects of financial receipt of JSY on immunization outcomes. The biggest drops in effect size were among women delivering in a health facility, as would be expected, given the pathway of increased immunization as a result of interaction with a health facility is not being captured.

The evolution of JSY post 2008 was rapid. The program has expanded considerably since it began in 2005, reaching over 10 million beneficiaries in 2011-2012 (up from 3 million in 2006–2007, and 7 million in 2007–2008).[35] Our results must therefore be interpreted in light of the current shape of the program, as well as other related programs that have more recently been implemented. With over 870,000 ASHAs currently engaged in communities in all states, and along with the recent home based newborn care scheme, an even bigger emphasis may be placed on childhood immunizations. Another new initiative launched in 2011, Janani Shishu Suraksha Karyakaram (JSSK), provides free and cashless services for delivery care in public institutions, including cesarean section, and postnatal care for sick newborns, and is being further expanded to include free antenatal and postnatal care for all infants.[36]

Despite the limitations, our findings have a number of promising implications. Increased childhood vaccination coverage as a result of JSY translates into protection from disease, disability and death among many children who would not have previously been immunized. Further insights gained from this analysis are the existence of untapped opportunities to piggyback additional benefits on to this program, such as improvements in breastfeeding behaviors, IMCI indicators, and nutrition and sanitation outcomes.

Vaccination is one of the most cost-effective ways to prevent disease and disability and improve childhood survival. However from an operational standpoint, increasing the coverage of immunizations can be difficult and costly. A pilot project conducted in Moradabad district of Uttar Pradesh from mid-2006 to early 2007 that sought to identify and vaccinate all newborns with oral polio vaccine within 72 hours of birth had disappointing results.[37] Researchers found the program to have high expansion costs and marginal impacts. One of the major insights from that study was that no mechanism was in place to routinely identify newborns, especially for deliveries that occur outside of health facilities.[37]

Janani Suraksha Yojana is one of the largest cash transfer programs in the world,[36] and offers a potential new opportunity to reach newborns and infants that previously would not have had much interaction with the health system. At an expenditure that increased from 383 million Rs. (∼$6.3 million) in the 2004–2005 financial year to 16 billion Rs. (∼$266 million in 2011–2012),[38] policy makers must be aware of the financial implications of the program. Although we have not attempted to estimate the cost-effectiveness of India's JSY program here, this is an important area of further research.[21]

The structuring of financial incentives also requires careful consideration. Early assessments of JSY point to delays in receipt of payments by mothers and ASHAs, and in some cases, informal payments were required to receive the cash.[15], [39] Grievances by ASHAs regarding the uneven balance between expected workload and payment received for some services (including immunizations) could indicate the need for a revision of the payment structure.[40] The home-based newborn care and home delivery of contraceptives schemes will help to increase the incentives ASHAs receive every month and could help allay the grievances of ASHAs. Recent evidence of corruption in India's most populous state, Uttar Pradesh, which has some of the worst health indicators and therefore was also the state allocated the largest budget for JSY, warns of the need for systems in place to monitor and evaluate the scheme carefully at all levels of administration.[41]

There are important health systems issues that could jeopardize the success of the program. Shortages of human resources and absence of health personnel in facilities are problematic, as these workers are needed to administer vaccines. There is also substantial evidence of poor quality of infrastructure, including limited cold chain capacity of many states for accommodating even routine UIP vaccines, and limited awareness in some areas about safe injection practices and waste management.[42] Further monitoring of vaccine coverage is critical, and additional health systems research to identify and target poor management, and lack of human resources, infrastructure and supplies is necessary. Behavioral research into the role of ASHAs and their influence on reproductive health behaviors is also a priority. Finally, given the persistent health and coverage inequalities across geographic areas socioeconomic groups in India, it will be essential to ensure the program reaches population groups that were initially targeted as having the highest need.

In December 2010, a Decade of Vaccines Collaboration was declared by a partnership of international agencies working in immunization.[7] While India is still far off from achieving 90% coverage of DPT3, one of the goals of the Global Immunization Vision and Strategy, it is evidently progressing in the right direction.[7] With one fifth of all children under-five in the world,[43] even a few percentage points increase in childhood immunization rates could be of global health significance. India has achieved success in its polio eradication efforts, with 2011 being the first year India was declared polio free; policy makers must sustain efforts to preserve these successes.

## Supporting Information

Table S1 Background characteristics* of women, pre- and post-matching. * Shown for the Fully-vaccinated child outcome only; restricted to women with children aged 12 to 24 months.
(DOCX)

Table S2 Regression results for immunization outcomes, run on sample of children 12-23 months of age.
(DOCX)

Table S3 Immunization coverage estimates by immunization card possession, most recent births 12-23 months prior to survey to women 15-44 years. *A fully vaccinated child was defined as a child who had received one dose of BCG vaccine, 3 doses of DPT and polio vaccines (not including polio at birth), and one dose of measles vaccine. [IIPS 2010]
(DOCX)

Figure S1 Histograms* of propensity scores. * Shown for the fully vaccinated child outcome only.
(TIFF)

Figure S2 **Estimated JSY treatment effect on childhood immunization outcomes among children 12 to 23 months of age: Propensity Score Matching compared with Coarsened Exact Matching.** Error bars represent 95% confidence intervals from regression estimates. * A fully vaccinated child was defined as a child who had received one dose of BCG vaccine, 3 doses of DPT and polio vaccines (not including polio at birth), and one dose of measles vaccine. [IIPS 2010]
(TIFF)
