# Supplementary material for: More Evidence on the Impact of India's Conditional Cash Transfer Program, Janani Suraksha Yojana: Quasi-Experimental Evaluation of the Effects on Childhood Immunization and Other Reproductive and Child Health Outcomes
Source: PLoS One. 2014 Oct 10;9(10):e109311. doi: 10.1371/journal.pone.0109311 (PMC4193776; doi:10.1371/journal.pone.0109311)
Supplement: Table S2 — Regression results for immunization outcomes, run on sample of children 12-23 months of age. (DOCX) [file pone.0109311.s002.docx]

***Appendix Table 2:* Regression results for immunization outcomes, run on sample of children 12-23 months of age (Continued)**
