# Supplementary material for: More Evidence on the Impact of India's Conditional Cash Transfer Program, Janani Suraksha Yojana: Quasi-Experimental Evaluation of the Effects on Childhood Immunization and Other Reproductive and Child Health Outcomes
Source: PLoS One. 2014 Oct 10;9(10):e109311. doi: 10.1371/journal.pone.0109311 (PMC4193776; doi:10.1371/journal.pone.0109311)
Supplement: Table S3 — Immunization coverage estimates by immunization card possession, most recent births 12-23 months prior to survey to women 15-44 years. *A fully vaccinated child was defined as a child who had received one dose of BCG vaccine, 3 doses of DPT and polio vaccines (not including polio at birth), and one dose of measles vaccine. [IIPS 2010] (DOCX) [file pone.0109311.s003.docx]

|  | With immunization card | | | Without immunization card | | |
| --- | --- | --- | --- | --- | --- | --- |
|  | Mean | 95% CI, upper | 95% CI, lower | Mean | 95% CI, upper | 95% CI, lower |
| BCG | 98.9% | 98.7% | 99.0% | 78.7% | 78.2% | 79.1% |
| Polio at birth | 81.8% | 81.3% | 82.3% | 59.1% | 58.5% | 59.7% |
| Polio 1 | 97.7% | 97.6% | 97.9% | 90.7% | 90.3% | 91.0% |
| Polio 3 | 89.4% | 89.0% | 89.8% | 54.6% | 54.0% | 55.3% |
| DPT 1 | 98.6% | 98.4% | 98.8% | 72.3% | 71.7% | 72.8% |
| DPT 3 | 90.2% | 89.8% | 90.6% | 46.1% | 45.5% | 46.7% |
| Measles | 83.1% | 82.6% | 83.6% | 61.2% | 60.6% | 61.8% |
| Hepatitis B | 37.2% | 36.5% | 37.9% | 23.6% | 23.0% | 24.1% |
| Fully vaccinated child* | 80.6% | 80.1% | 81.2% | 33.7% | 33.1% | 34.2% |
| No vaccine | 0.1% | 0.1% | 0.1% | 8.1% | 7.8% | 8.5% |
