# Supplementary figures and images for: More Evidence on the Impact of India's Conditional Cash Transfer Program, Janani Suraksha Yojana: Quasi-Experimental Evaluation of the Effects on Childhood Immunization and Other Reproductive and Child Health Outcomes
Source: PLoS One. 2014 Oct 10;9(10):e109311. doi: 10.1371/journal.pone.0109311 (PMC4193776; doi:10.1371/journal.pone.0109311)

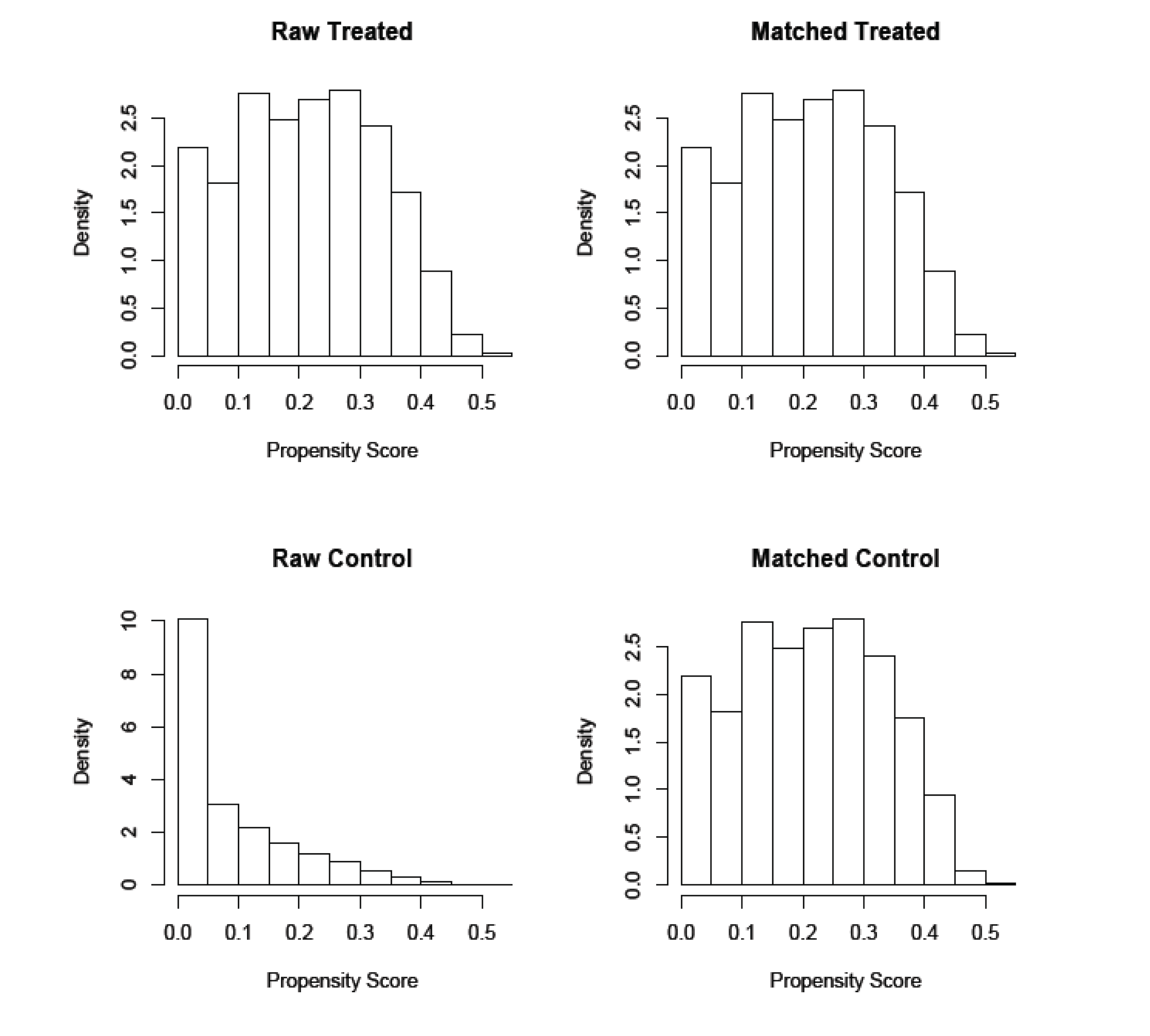

Supplement: Figure S1 — Histograms* of propensity scores. * Shown for the fully vaccinated child outcome only. (TIFF) [file pone.0109311.s004.tiff]

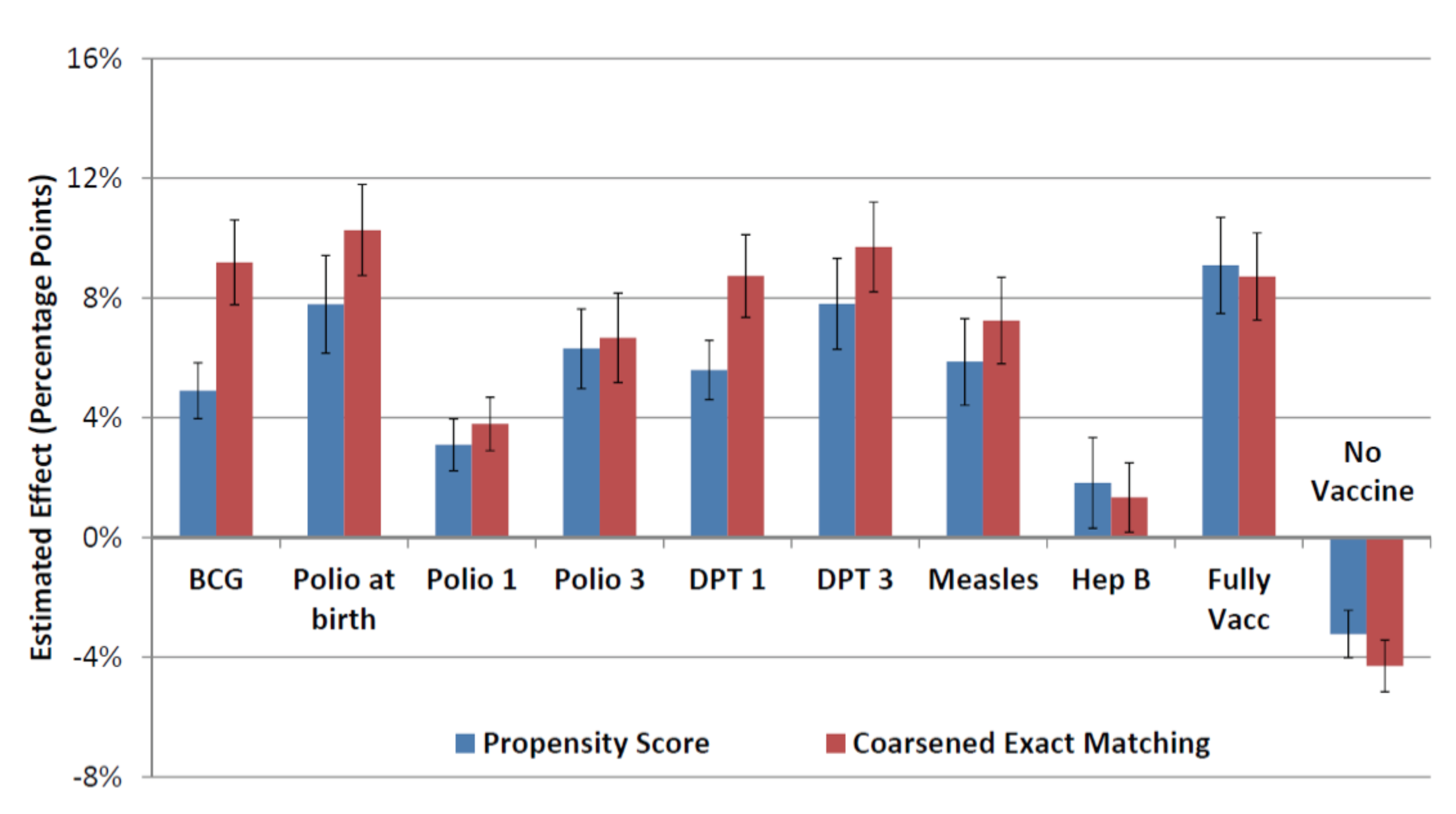

Supplement: Figure S2 — Estimated JSY treatment effect on childhood immunization outcomes among children 12 to 23 months of age: Propensity Score Matching compared with Coarsened Exact Matching. Error bars represent 95% confidence intervals from regression estimates. * A fully vaccinated child was defined as a child who had received one dose of BCG vaccine, 3 doses of DPT and polio vaccines (not including polio at birth), and one dose of measles vaccine. [IIPS 2010] (TIFF) [file pone.0109311.s005.tiff]
