# Supplementary material for: More Evidence on the Impact of India's Conditional Cash Transfer Program, Janani Suraksha Yojana: Quasi-Experimental Evaluation of the Effects on Childhood Immunization and Other Reproductive and Child Health Outcomes
Source: PLoS One. 2014 Oct 10;9(10):e109311. doi: 10.1371/journal.pone.0109311 (PMC4193776; doi:10.1371/journal.pone.0109311)
Supplement: Supplementary file 4 [file pone.0109311.s001.docx]

		UNMATCHED DATA					
		JSY = 0 (n = 54,162)		JSY = 1 (n = 6,435)		Total (n = 62,981)	
		No.	%	No.	%	No.	%
Maternal age (years)	**15-19**	3,415	6.3	514	8	3,929	6.5
	**20-24**	20,265	37.4	2,971	46.2	23,236	38.3
	**25-29**	18,020	33.3	2,012	31.3	20,032	33.1
	**30-34**	8,090	14.9	673	10.5	8,763	14.5
	**35-39**	3,235	6	213	3.3	3,448	5.7
	**40-44**	933	1.7	45	0.7	978	1.6
Number of live births	**1 birth**	15,616	28.8	2,707	42.1	18,323	30.2
	**2 births**	14,690	27.1	2,204	34.3	16,894	27.9
	**3-4 births**	18,912	34.9	1,325	20.6	20,237	33.4
	**5 or more**	4,942	9.1	199	3.1	5,141	8.5
Maternal education	**No education**	25,272	46.7	2,070	32.2	27,342	45.1
	**1 to 5 years**	7,834	14.5	1,125	17.5	8,959	14.8
	**6 to 11 years**	15,604	28.8	2,649	41.2	18,253	30.1
	**12 years or more**	5,430	10	589	9.2	6,019	9.9
Caste	**Scheduled caste**	10,260	18.9	1,408	21.9	11,668	19.3
	**Scheduled tribe**	9,048	16.7	1,363	21.2	10,411	17.2
	**Other backward class**	22,155	40.9	2,444	38	24,599	40.6
	**Other**	12,699	23.4	1,220	19	13,919	23
Religion	**Hindu**	40,961	75.6	5,284	82.1	46,245	76.3
	**Muslim**	8,101	15	593	9.2	8,694	14.3
	**Christian**	2,552	4.7	396	6.2	2,948	4.9
	**Sikh**	1,118	2.1	29	0.5	1,147	1.9
	**Buddhist**	496	0.9	99	1.5	595	1
	**Other**	934	1.7	34	0.5	968	1.6
Distance to facility (kms)	**0 - 4**	38,827	72.1	4,913	76.7	43,740	72.6
	**5 - 9**	6,435	12	680	10.6	7,115	11.8
	**10 - 19**	5,678	10.5	524	8.2	6,202	10.3
	**20+**	2,898	5.4	290	4.5	3,188	5.3
Location	**Rural**	44,090	81.4	5,310	82.5	49,400	81.5
	**Urban**	10,072	18.6	1,125	17.5	11,197	18.5
Wealth quintile	**1**	11,546	21.3	1,064	16.5	12,610	20.8
	**2**	11,979	22.1	1,235	19.2	13,214	21.8
	**3**	9,487	17.5	1,373	21.3	10,860	17.9
	**4**	10,571	19.5	1,690	26.3	12,261	20.2
	**5**	10,578	19.5	1,073	16.7	11,651	19.2

		PROPENSITY SCORE MATCHED DATASET					
		JSY = 0 (n = 6,336)		JSY = 1 (n = 6,336)		Total (n = 12,672)	
		No.	%	No.	%	No.	%
Maternal age (years)	**15-19**	515	8.1	506	8.0	1,021	8.1
	**20-24**	2,966	46.8	2,925	46.2	5,891	46.5
	**25-29**	1,946	30.7	1,989	31.4	3,935	31.1
	**30-34**	664	10.5	664	10.5	1,328	10.5
	**35-39**	201	3.2	209	3.3	410	3.2
	**40-44**	44	0.7	43	0.7	87	0.7
Number of live births	**1 birth**	2,682	42.3	2,656	41.9	5,338	42.1
	**2 births**	2,136	33.7	2,179	34.4	4,315	34.1
	**3-4 births**	1,323	20.9	1,307	20.6	2,630	20.8
	**5 or more**	195	3.1	194	3.1	389	3.1
Maternal education	**No education**	2,036	32.1	2,028	32	4,064	32.1
	**1 to 5 years**	1,095	17.3	1,109	17.5	2,204	17.4
	**6 to 11 years**	2,599	41	2,613	41.3	5,212	41.1
	**12 years or more**	604	9.5	584	9.2	1,188	9.4
Caste	**Scheduled caste**	1,429	22.6	1,385	21.9	2,814	22.2
	**Scheduled tribe**	1,319	20.8	1,324	20.9	2,643	20.9
	**Other backward class**	2,406	38	2,420	38.2	4,826	38.1
	**Other**	1,182	18.7	1,207	19	2,389	18.9
Religion	**Hindu**	5,224	82.4	5,224	82.4	10,448	82.4
	**Muslim**	581	9.2	583	9.2	1,164	9.2
	**Christian**	362	5.7	367	5.8	729	5.8
	**Sikh**	34	0.5	29	0.5	63	0.5
	**Buddhist**	101	1.6	99	1.6	200	1.6
	**Other**	34	0.5	34	0.5	68	0.5
Distance to facility (kms)	**0 - 4**	4,851	76.9	4,832	76.6	9,683	76.8
	**5 - 9**	678	10.8	675	10.7	1,353	10.7
	**10 - 19**	500	7.9	517	8.2	1,017	8.1
	**20+**	276	4.4	284	4.5	560	4.4
Location	**Rural**	5,248	82.8	5,233	82.6	10,481	82.7
	**Urban**	1,088	17.2	1,103	17.4	2,191	17.3
Wealth quintile	**1**	1,130	17.8	1,045	16.5	2,175	17.2
	**2**	1,255	19.8	1,210	19.1	2,465	19.5
	**3**	1,198	18.9	1,352	21.3	2,550	20.1
	**4**	1,635	25.8	1,669	26.3	3,304	26.1
	**5**	1,117	17.6	1,060	16.7	2,177	17.2
